# Co-culture with periodontal ligament stem cells enhances osteogenic gene expression in de-differentiated fat cells

**DOI:** 10.1007/s13577-014-0091-1

**Published:** 2014-02-27

**Authors:** Kallapat Tansriratanawong, Yuichi Tamaki, Hiroshi Ishikawa, Soh Sato

**Affiliations:** 1 Department of NDU Life Sciences, Nippon Dental University School of Life Dentistry at Tokyo, 1-9-20 Fujimi, Chiyoda-ku, Tokyo, 102-8159 Japan; 2 Department of Periodontology, Nippon Dental University School of Life Dentistry at Niigata, 1-8 Hamaura-cho, Chuo-ku, Niigata, 951-1500 Japan; 3 Department of Oral Medicine and Periodontology, Faculty of Dentistry, Mahidol University, 6 Yothi Street Rajthevi, Bangkok, 10400 Thailand; 4 Department of Developmental and Regenerative Dentistry, Nippon Dental University School of Life Dentistry at Tokyo, 1-9-20 Fujimi, Chiyoda-ku, Tokyo, 102-8159 Japan

**Keywords:** Co-culture, PDLSCs, DFAT cells, Osteogenesis, DNA methylation

## Abstract

In recent decades, de-differentiated fat cells (DFAT cells) have emerged in regenerative medicine because of their trans-differentiation capability and the fact that their characteristics are similar to bone marrow mesenchymal stem cells. Even so, there is no evidence to support the osteogenic induction using DFAT cells in periodontal regeneration and also the co-culture system. Consequently, this study sought to evaluate the DFAT cells co-culture with periodontal ligament stem cells (PDLSCs) in vitro in terms of gene expression by comparing runt-related transcription factor 2 (RUNX2) and Peroxisome proliferator-activated receptor gamma 2 (PPARγ2) genes. We isolated DFAT cells from mature adipocytes and compared proliferation with PDLSCs. After co-culture with PDLSCs, we analyzed transcriptional activity implying by DNA methylation in all adipogenic gene promoters using combined bisulfite restriction analysis. We compared gene expression in RUNX2 gene with the PPARγ2 gene using quantitative RT-PCR. After being sub-cultured, DFAT cells demonstrated morphology similar to fibroblast-like cells. At the same time, PDLSCs established all stem cell characteristics. Interestingly, the co-culture system attenuated proliferation while enhancing osteogenic gene expression in RUNX2 gene. Using the co-culture system, DFAT cells could trans-differentiate into osteogenic lineage enhancing, but conversely, their adipogenic characteristic diminished. Therefore, DFAT cells and the co-culture system might be a novel cell-based therapy for promoting osteogenic differentiation in periodontal regeneration.

## Introduction

The ultimate goal of periodontal therapy is periodontal regeneration, which is defined as the establishment and reconstruction of new periodontium into functional architectures using lost or injured tissues [1, 2]. While we used several procedures to achieve regeneration, stem cells produced the best outcomes. Dental stem cells are categorized in cell-based procedures for tissue engineering. They derived from dental organs, including dental pulp, periodontal ligament, root apical papilla, and dental follicle, which possess a high potential for use in regenerative medicine [3–6]. They share characteristics similar to mesenchymal stem cells (MSCs) in terms of their colony-forming efficiency, proliferation, and multi-lineage differentiation. Among these, periodontal ligament stem cells (PDLSCs) have shown potential in osteogenic differentiation for periodontal regeneration and have also exhibited multi-lineage differentiation into adipogenic, chondrogenic, and neurogenic lineage [7–9]. It was possible to isolate PDLSCs from the heterogeneous population in periodontal ligaments, which contained varieties of progenitor cells and differentiating cells, using single colony cloning and stem cell markers sorting. They expressed the surface stem cell antigen markers, which included CD44, CD73, CD90, CD105, CD146, and Stro-1 [5, 10, 11].

Beyond dental stem cells, alternative sources for cell-based therapy for periodontal regeneration have gained more attention in somatic stem cells. Recently, de-differentiated fat cells (DFAT cells) emerged as a possible alternative cell source for regeneration. Subcutaneous fat tissue can be harvested easily and sufficiently obtained in appropriate amount for regenerative defects as compared to PDLSCs. Successfully isolating them using the ceiling method demonstrated characteristics similar to bone marrow mesenchymal stem cells (BMMSCs) and adipose-derived stem cells (ASCs) [12–15]. Moreover, much evidence showed that DFAT cells could trans-differentiate into other cell types, such as cardiocytes [16], smooth muscle-like cells [17], and urethral sphincter cells [18]. While the role of DFAT cells for periodontal regeneration and their potential for osteogenic differentiation nevertheless remains unclear, a recent study hypothesized that DFAT cells might provide the trans-differentiation property for osteogenic differentiation for periodontal regeneration.

A co-culture system was used for cell culture improvement, mechanism investigation, and cell–cell interaction simulation, taking place between endothelial cells with MSCs, including in PDLSCs. A previous study demonstrated the signaling pathway of COX-2/PGE2/VEGE under the hypoxia condition up-regulating in osteogenic differentiation of PDLSCs after co-cultured with endothelial cells [19]. Moreover, a co-culture of endothelial cells with DFAT cells appeared to reverse the stemness characteristic and morphology similar to immature adipocytes [20]. Recently, a co-culture with MSCs performed in oral cells type provided immature features by expressing stem cell-associated genes [21]. Thus, we supposed that co-culture system with PDLSCs might not only simulate the periodontal environment but also provide the enhancement in osteogenic induction for DFAT cells.

Osteogenic and adipogenic lineages are recognized as the contrary lineage in MSCs differentiation [22]. Particularly, in runt-related transcription factor 2 (RUNX2) and Peroxisome proliferator-activated receptor gamma 2 (PPARγ2), they are proposed for flavor regulation in their osteogenic or adipogenic differentiation. We used DFAT cells, which are representative of the adipogenic lineage, and focused on whether DFAT cells might trans-differentiate into osteogenic differentiation and up-regulate the osteogenic gene expression.

We hypothesized that a co-culture of DFAT cells with PDLSCs might simulate the periodontal environment in vitro and enhance the osteogenic differentiation function for periodontal regeneration. This study aimed to evaluate the osteogenic gene expression of DFAT cells after being co-cultured with PDLSCs by detecting the RUNX2 gene expression level and comparing its effect to the PPARγ2 gene.

## Materials and methods

### Isolation and culture of DFAT cells

The protocol was approved by the ethics committees of Nippon Dental University (NDU-T2011-32). Subcutaneous adipose tissues were obtained from three healthy female subjects (58–85 years), who were given their written informed consents. Adipose tissues were isolated in accordance with ceiling method procedure [12]. Tissues were digested with warmed 3 mg/mL collagenase type I (Sigma, St Louis, MO) and 4 mg/mL dispase (Sanko Pure Chemical Ltd., Tokyo, Japan) at 37 °C for 1 h and subsequently centrifuged at 300×*g* for 15 min. Mature fat cells at the uppermost portion were collected following by incubating with erythrocyte lysis buffer at 4 °C for 15 min. Cell suspensions were then filtered through 70-μm nylon cell strainers (Falcon, BD Labware, Franklin Lakes, NJ) and seeded approximately 1 × 10^5^ cells in each 25-cm^2^ culture flask (NUNC, Roskilde, Denmark), which completely filled with growth medium (GM). Dulbecco’s modified Eagle’s medium/Ham’s nutrient mixture F12 (Gibco BRL, Carlsbad, CA) supplemented with 15 % fetal bovine serum (Gibco), 2 mM glutamine (GlutaMAX I, Invitrogen, Carlsbad, CA), 50 U/mL penicillin and 50 μg/mL streptomycin (Gibco BRL) were used as GM. Mature fat cells floated and attached to the upper surface of the flask. Then, flask was inverted with reduction the medium at 7–10 days. For cell morphology investigation, DFAT cells were rinsed with phosphate buffer saline (PBS) followed by fixed in 10 % formalin solution, and stained with Oil Red O (Wako). On the other hand, DFAT cells culture, which reached to confluence, were then sub-cultured by adding 0.1 % trypsin and 0.02 % ethylenediaminetetraacetic acid (EDTA)/PBS and split at 1:3 dilution in fresh medium.

### Isolation and culture of PDLSCs and BMMSCs

The periodontal ligaments at middle one-third of the impacted or premolar tooth roots from three healthy female subjects (17–25 years) were harvested and cut into small pieces following digested by enzyme. Isolation protocol was followed as described earlier [7]. Cell suspension was filtered through 70 μm nylon cell strainer and then, centrifugation was performed at 300×*g* for 15 min. Cells were retrieved in GM and approximately 1 × 10^4^ cells were seeded in each 100-mm dish (Nunc) as primary culture. For BMMSCs, three cell lines from passage (P) 3 were used as a control of MSCs [7].

### Population doubling time (PDT)

For determination of proliferative function, DFAT cells and PDLSCs were seeded at cell density of 1 × 10^4^ cells into 35-mm dish (Falcon). The numbers of cells were counted in triplicate every 2 days for 2 weeks. PDT was calculated by PDT software [40].

### Flow cytometric analysis

PDLSCs from P3 were harvested by trypsinization and split approximately 5 × 10^5^ cells per tube. Mouse monoclonal anti-human antibodies conjugated with fluorescein isothiocyanate-conjugated (FITC) and phycoerythrin (PE) were performed as follow: anti-CD-90-PE, anti-CD105-PE, anti-CD106-PE, and isotype control using immunoglobulin G (all from BD Biosciences, San Jose, CA); anti-CD-34-FITC, and anti-CD-44-FITC (Beckman coulter). Each aliquot was incubated in the dark at 4 °C for 20 min. Cell pellets were washed with PBS and resuspended in 1 % BSA/PBS. Flow cytometric analysis was performed in triplicate and determined in quantitative data using Guava Express Plus version 5.3 software (Guava Technology).

### Multilineage differentiation

PDLSCs were plated at density 1 × 10^4^ cells per well in 6-well plate. Once PDLSCs reached to the confluence, each differentiation medium was then substituted. Osteogenic differentiation was supplemented with 100 nM dexamethasone, 50 μM ascorbic acid, and 10 mM β-glycerophosphate. Adipogenic differentiation was supplemented with 1 μM dexamethasone, 0.5 mM isobutylmethylxanthine (IBMX), and 100 μM indomethacin. Chondrogenic differentiation was supplemented with 10 ng/mL transforming growth factor beta-1 (TGF-β1), 100 nM dexamethasone, 37.5 μg/mL ascorbic acid, 1 % insulin-transferrin-selenium (ITS), and 1 mM sodium pyruvate. All lineage differentiations were cultured for 3 weeks subsequently by fixation with 10 % formalin solution and stained as follows: osteogenic differentiation was stained by 1 % Alizarin Red (Certistain^®^, Darmstadt, Germany) at pH 4.2 for 30 min, adipogenic differentiation was stained by Oil Red O, and chondrogenic differentiation was stained by 0.1 % Toluidine Blue (Muto Pure Chemical, Japan), respectively.

### Reverse transcriptase polymerase chain reaction (RT-PCR)

Multilineage differentiation was confirmed genes expression by RT-PCR. Total RNA was extracted using RNeasy Mini kit (Qiagen, Hilden, Germany) according to the manufacturer’s protocol and determined quantity of RNA by 260/280 nm absorbance. cDNA was synthesized from 1 μg of RNA using the High Capacity cDNA synthesis kit (Applied Biosystems, Carlsbad, CA). The PCR Supermix Platinum kit (Invitrogen) was used for amplification following by condition of preincubation at 94 °C for 2 min, then performed by 35 cycles of denaturation at 94 °C for 30 s; primer annealing at 52–60 °C for 30 s and extension step at 72 °C for 1 min. Finally, a post extension step was done at 72 °C for 7 min. PCR products were electrophoresed using 2 % agarose gel being stained with 0.5 μg/mL ethidium bromide (EtBr). RT-PCR primers are listed in the Table 1. GAPDH was used as an endogenous control.

**Table 1:** Primers used for RT-PCR and quantitative RT-PCR

Gene	Oligonucleotide sequence		Product size	GenBank No.	Annealing temperature (°C)
	Forward primer	Reverse primer	(bp)		
RUNX2	5′-CCCCACGACAACCGCACCAT-3′	5′-GTCCACTCCGGCCCACAAATC-3′	292	NM_004348	60
Alkaline phosphatase	5′-AACATCAGGGACATTGACGTG-3′	5′-GTATCTCGGTTTGAAGCTCTTCC-3′	159	NM_001127501	57
LPL	5′-GAGATTTCTCTGTATGGCACC-3′	5′-CTGCAAATGAGACACTTTCTC-3′	276	NM_000237	52
PPARγ2	5′-GGGATCAGCTCCGTGGATCT-3′	5′-TGCACTTTGGTACTCTTGAAGTT-3′	186	NM_138711	60
COL2A1	5′-TTCAGCTATGGAGATGACAATC-3′	5′-AGAGTCCTAGAGTGACTGAG-3′	472	NM_001844	60
Sox9	5′-GGTTGTTGGAGCTTTCCTCA-3′	5′-TAGCCTCCCTCACTCCAAGA-3′	401	NM_000346.3	52
GAPDH	5′-GTCAAGGCTGAGAACGGGAA-3′	5′-GCTTCACCACCTTCTTGATG-3′	613	NM_001256799.1	55
β-actin	5′-AGAGCTACGAGCTGCCTGAC-3′	5′-AGCACTGTGTTGGCGTACAG-3′	184	NM_001101.3	60

### Co-culture system

For construction of cell–cell interactive environment, DFAT cells and PDLSCs from P3 were used. DFAT cells were plated at the density 1 × 10^4^ cells/well in 6-well plate (Sumilon, Sumitomo, Japan). On the contrary, PDLSCs were plated 1 × 10^3^ cells/well in the 0.4 μm pore size of 6-transwell insertion (Falcon). Co-culture DFAT cells with PDLSCs were extended for 2 weeks followed by DNA extraction and methylation analysis. On the other hand, for determining osteogenic gene expression, co-culture was continuously cultured for further 2 weeks replacing by osteogenic differentiation medium as described earlier in multilineage differentiation. Non-co-culture group was defined DFAT cells culture without PDLSCs.

### DNA extraction and bisulfite conversion

Genomic DNA was extracted from co-culture, non-co-culture, and BMMSCs group using the DNeasy^®^ Blood & Tissue kit (Qiagen). Cells were digested by lysis buffer from manufacturer and isolated DNA aliquot. Then, bisulfite modification was performed to DNA using the EpiTect^®^ bisulfite kit (Qiagen). In brief, 1 μg of DNA was mixed with the bisulfite mixture and carried out thermal cycler approximately 5 h. PCR was used for amplifying bisulfite modified DNA as follows: preincubation at 94 °C for 2 min, then performed by 40 cycles of denaturation at 94 °C for 1 min; primer annealing at 54–57 °C for 1 min, extension step at 65 °C for 1 min, and post extension step at 65 °C for 7 min using bisulfite primers sets for CCAAT/enhancer binding protein alpha (C/EBPα), Fatty acid binding protein 4 (FABP4), Lipoprotein lipase (LPL), PPARγ2, and RUNX2 gene promoters as shown in Table 2.

**Table 2:** Bisulfite primers

Gene	Oligonucleotide sequence		Product size (bp)	GenBank No.	Annealing temperature (°C)
	Forward primer	Reverse primer			
C/EBPα	5′-GTTTGGTTTTGGTTTTGAAAG-3′	5′-CCAACTTTTATACCCAACAAAC-3′	420	U34070.1	55
FABP4	5′-GGTAATTTTTGAGATAGGAGTGTTT-3′	5′-CCAATTAAAAATAAAATCCAATCATTT-3′	418	AC018616	57
LPL	5′-GGGAGGATTGTAAGTGATAAATAGG-3′	5′-CAACTAAAAATAAACAACTTTCCCTT-3′	457	X68111.1	57
PPARγ2	5′-GTTGAAGTTTTTAAGAAAGTAAATT-3′	5′-AAAAAAAATATTACCACACTATCTC-3′	480	AB005520.1	54
RUNX2	5′-GGGGGAAAAGTTATAGTGGTAG-3′	5′-AAACAAAAAAACAAAACAAAAAAA-3′	364	NG_008020.1	55

### Combined bisulfite restriction analysis (COBRA)

PCR products of bisulfite modified DNA from all groups were digested with 20 U of restriction enzymes overnight, which were specific in the restriction sites by *HpyCH4IV* (ACGT) for C/EBPα, LPL, PPARγ2 and *Taq I* (TCGA) for FABP4 and RUNX2 gene (New England Biolabs, Ipswich, MA, USA). The digested PCR products were electrophoresed using 2 % agarose gel and being stained with 0.5 μg/mL of EtBr. Each gene fragment length was shown as follows: C/EBPα, the amplicons provided 171, 249, and 420 base pair (bp): FABP4, the amplicons provided 56, 85, 141, 272, 357, and 413 bp: LPL, the amplicons provided 121, 164, 172, 285, 336, and 457 bp: PPARγ2, the amplicons provided 62, 181, 237, 299, 418, and 480 bp: and RUNX2, the amplicons provided 103, 261, and 364 bp. MultiGauge V3.0 software (Fujifilm, Japan) was analyzed each band intensity and methylation in percentage, which were calculated by following formula: methylation percentage = 100 × digested fragments/undigested fragments + digested fragments.

### Real-time PCR and semi-quantitative RT-PCR

Osteogenic differentiation potential from co-culture and non-co-culture group was compared in RUNX2 and PPARγ2 gene. The comparison of relative gene expression from RUNX2 and PPARγ2 gene was indicated by Power SYBR^®^ Green PCR Master Mix (Life Technologies, UK). The β-actin was used as an endogenous control. Real-time PCR primers are included in list of Table 1. For real-time PCR reaction, 500 ng of cDNA, 5 μM of each forward and reverse primer, 10 μL of SYBR Green, and distilled water were mixed in 96-well plate. The condition was performed preincubation at 95 °C for 10 min, followed by 40 cycles of denaturation at 95 °C for 15 s; primer annealing at 60 °C for 1 min and extension step at 95 °C for 15 s. Data were analyzed by StepOne™ software version 2.1. For semi-quantitative RT-PCR, serial concentrations were measured the band density using MultiGauge software. Relative band densities were calculated by normalization to GAPDH, which was used as an endogenous control.

### Statistical analysis

Data were reported as mean ±SD. Independent sample *t* test was used for analysis two-group comparison, whereas One-way ANOVA or Kruskal–Wallis test was used for the intergroup comparison. Differences at *P* < 0.05 were considered to be statistically significant. All experiments were performed in triplicate and were repeated with isolated cells from different subjects.

## Results

### DFAT cells can de-differentiate from mature fat cells to fibroblast-like cells

**Fig. 1:**
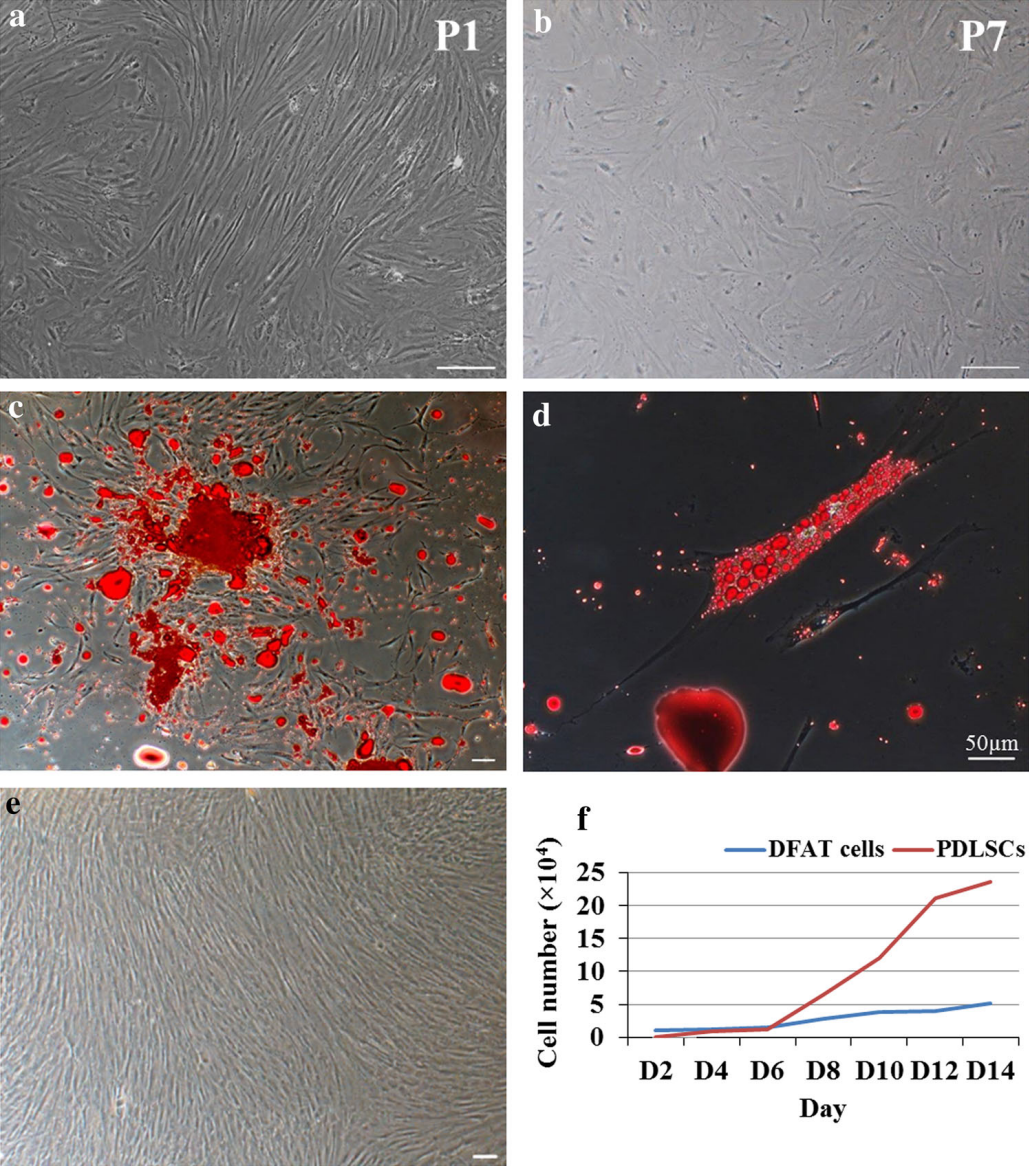
Morphologies and population doubling time comparison of DFAT cells and PDLSCs. **a**, **b** DFAT cells from passage 1 (P1) mostly demonstrated in fibroblast-like cells but gradually flatten and provided polyhedral morphology when cultured to the passage 7 (P7). DFAT cells, which were isolated from 7 to 10 days of primary culture, were positive stained lipid droplets by Oil Red O (**c**, **d**). PDLSCs exhibited fibroblast-like cells homogenously (**e**). Population doubling time (PDT) comparing between DFAT cells and PDLSCs was analyzed every 2 days for 2 weeks. PDLSCs provided shorter PDT, which implied as higher proliferation potential (**f**). *Scale bar* without character indicated 100 μm

DFAT cells were successfully isolated by the ceiling method, which could de-differentiate and proliferate from mature fat cells to fibroblast-like cells. In this study, DFAT cells displayed high proliferation potential after sub-cultured of primary culture. However, morphology gradually changed to all polyhedral shape in P7 (Fig. 1a, b). From day 7 to 10 in primary culture, DFAT cells demonstrated morphologic heterogeneity including fibroblast-like cells, polyhedral cells, and cell containing lipid droplets, which positively stained by Oil Red O (Fig. 1c, d). On the other hand, PDLSCs appeared in all fibroblast-like cells and actively expanded (Fig. 1e).

### PDLSCs exhibit higher proliferation than DFAT cells

PDLSCs and DFAT cells were compared the proliferative function in PDT for 2 weeks. PDLSCs exhibited approximately 2 times shorter in PDT (2.62) when compared with DFAT cells (5.04) (Fig. 1f).

### Stem cell characterizations of PDLSCs

**Fig. 2:**
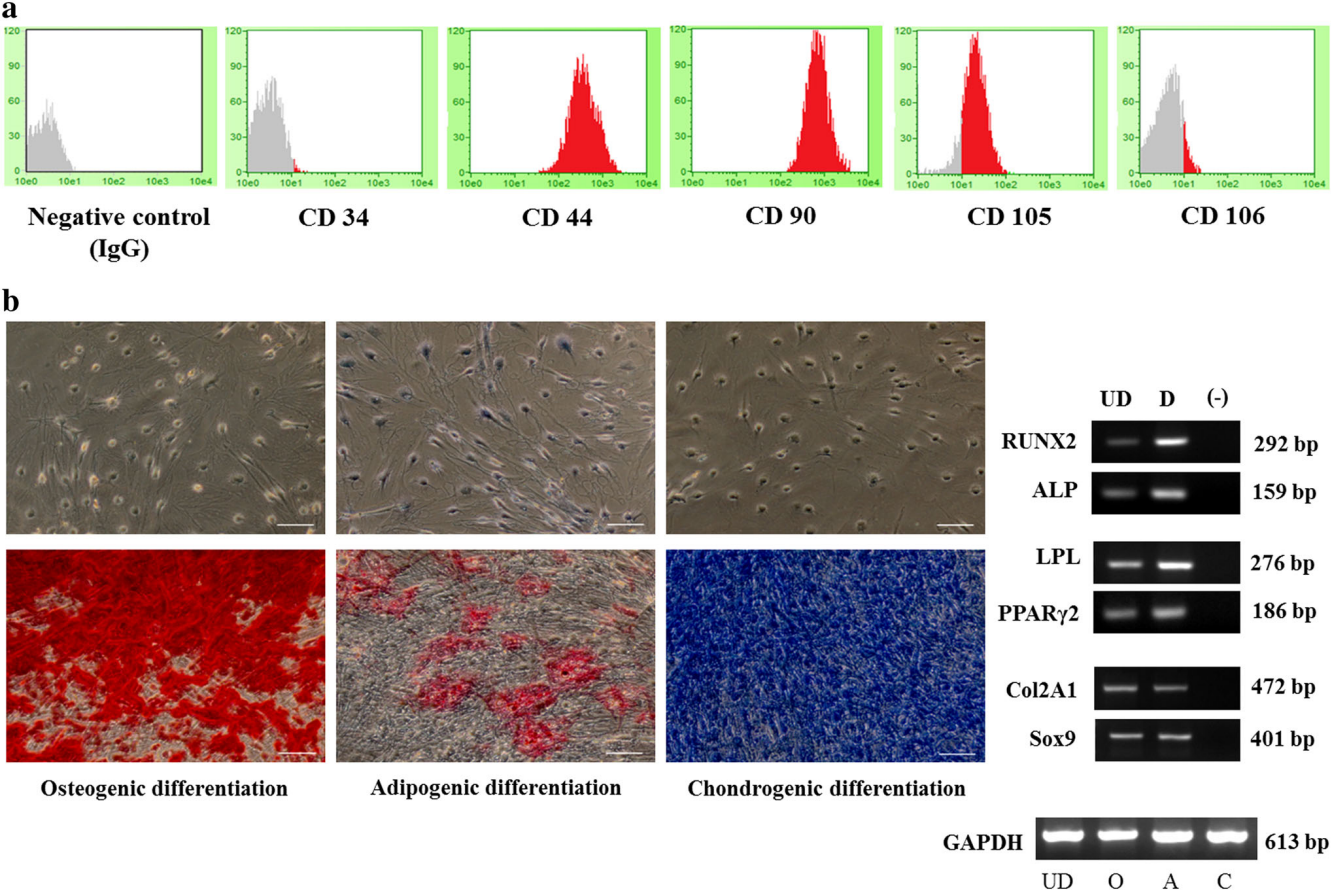
Stem cells characterizations of PDLSCs. **a** Flow cytometric analysis was performed for detecting immunophenotypes, which displayed all mesenchymal stem cell markers: CD44, CD90, CD105 but negatively shown the vascular cell markers: CD34 and CD106. Immunoglobulin G was used as a negative control, which demonstrated in all grey filled. Positive cell expressions were present by red filled. **b** PDLSCs were successfully induced into osteogenic (Alizarin Red staining), adipogenic (Oil Red O staining), and chondrogenic (Toluidine Blue staining) differentiation, which were confirmed gene expression of each lineage by RT-PCR. GAPDH was used as endogenous control. UD means undifferentiated PDLSCs, (−) means negative control. O means osteogenic-differentiated PDLSCs. A means adipogenic-differentiated PDLSCs. C means chondrogenic-differentiated PDLSCs. bp means base pairs. Scale bar indicated 100 μm

To confirm stem cell characteristics of PDLSCs, immunophenotypes by flow cytometry, and multilineage differentiation were performed. PDLSCs demonstrated cell surface antigen markers of MSCs (mean percentage ±SD, *n* = 3), including CD90 (99.93 ± 0.1), CD105 (85.66 ± 0.06), and adherence cell marker, CD44 (98.78 ± 1.73); in contrast, CD34, which was a hematopoietic stem cell marker, was negatively found (0.26 ± 0.13). CD106, which is the vascular adhesion molecule (VCAM-1), was detected from PDLSCs approximately 8.15 ± 0.36. All surface antigen markers were compared to negative control using isotype IgG (Fig. 2a). PDLSCs were induced in three different differentiated media for multilineage differentiation, including osteogenic (Alizarin Red staining), adipogenic (Oil Red O staining), and chondrogenic (Toluidine Blue staining) differentiation for 3 weeks. All lineages were confirmed genes expression by RT-PCR (Fig. 2b).

### Co-culture system up-regulates methylation status in all adipogenic gene promoters but down-regulates in osteogenic RUNX2 gene promoter

**Fig. 3:**
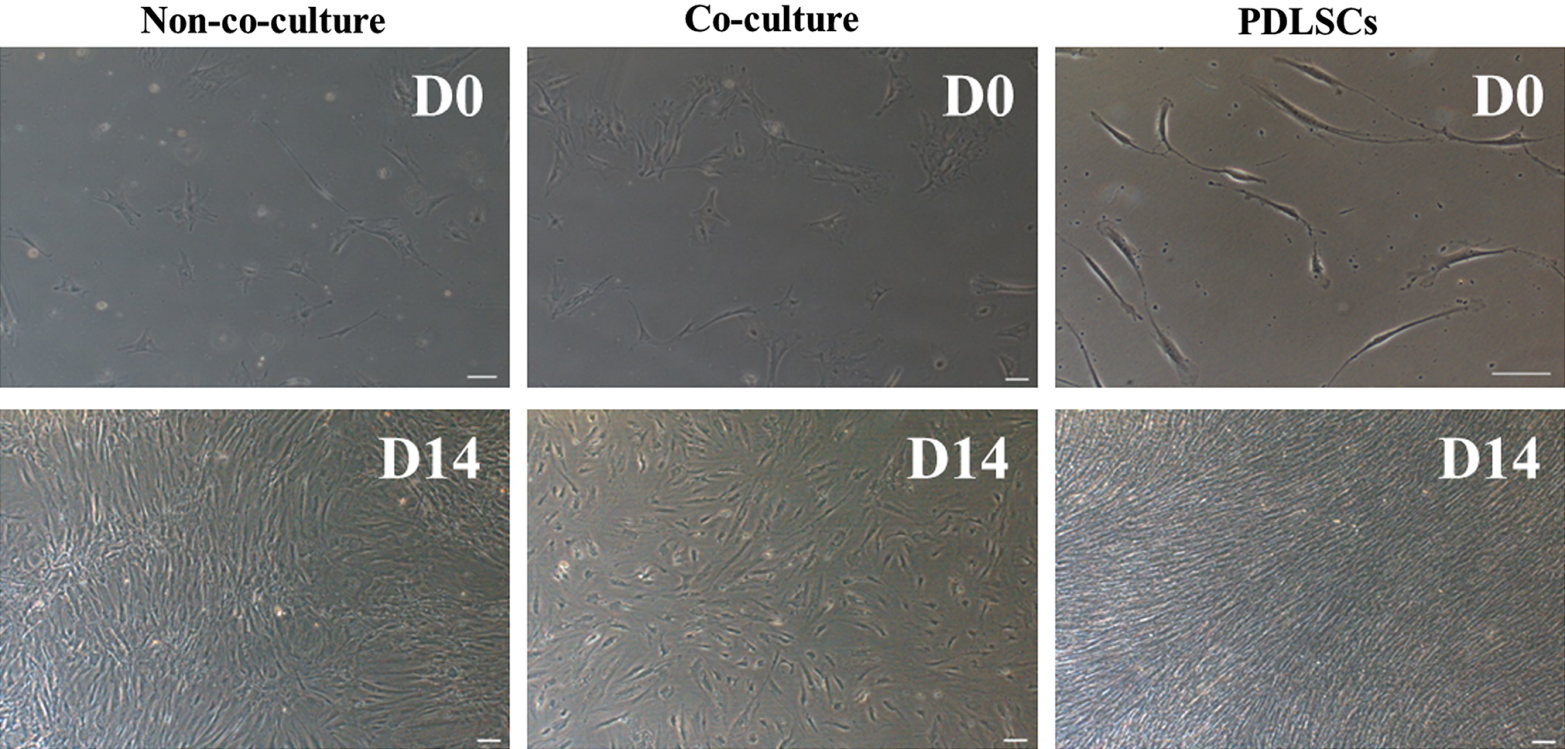
Cell morphology and distribution after co-cultured. Co-culture system was performed for 2 weeks followed by identifying cellular morphology. DFAT cells from co-culture group have dispersedly in cell distribution and shown more polyhedral shape comparing to non-co-culture group. Scale bar indicated 100 μm

**Fig. 4:**
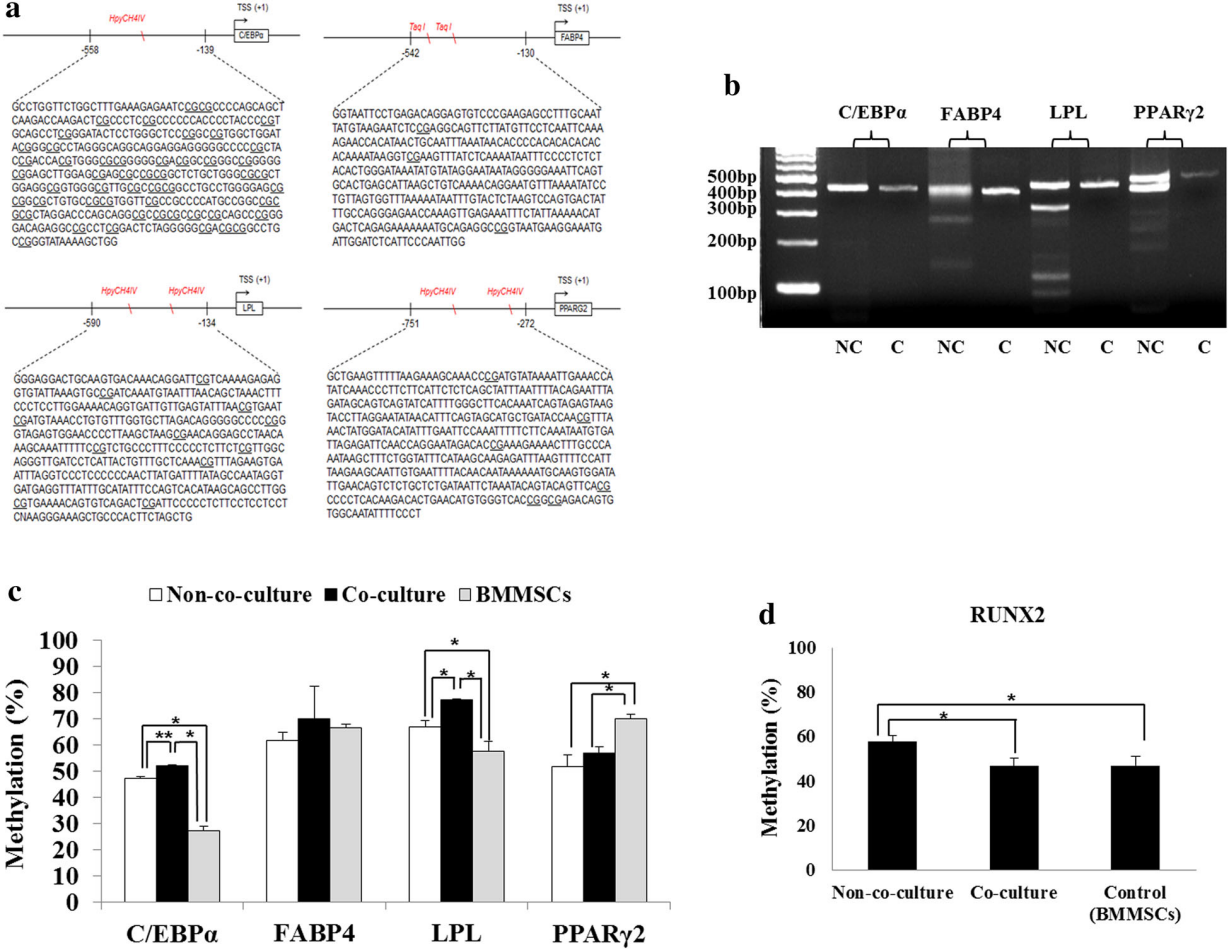
DNA methylation profiles of four adipogenic genes and RUNX2 gene by COBRA technique. (**a**, **b**) After co-cultured for 2 weeks, DNA methylation analysis of four adipogenic genes was analyzed by COBRA technique. Each PCR product was digested by restriction enzymes; *HpyCH4IV* (ACGT) for C/EBPα, LPL, PPARγ2 and *Taq I* (TCGA) for FABP4 and providing fragments as follows: C/EBPα gene (171, 249, and 420 bp), FABP4 gene (56, 85, 141, 272, 357, and 413 bp), LPL gene (121, 164, 172, 285, 336, and 457 bp), and PPARγ2 gene (62, 181, 237, 299, 418, and 480 bp). **c** Co-culture group demonstrated higher in methylation status in all adipogenic genes when compared with non-co-culture group, which implied for retardation in transcriptional activity. **d** RUNX2 gene has adversely demonstrated the methylation profile by showing lower methylation status after performed co-culture. NC means non-co-culture. *C* means co-culture, *bp* means base pairs. *Scale bar* indicated 100 μm. *Astrerisk* means that comparison was statistically significant difference at *P* < 0.05, *double asterisk* means that comparison was statistically significant difference at *P* < 0.01

DFAT cells were co-cultured with PDLSCs in 6-transwell plates for 2 weeks. Co-culture group demonstrated sparse cell distribution that mostly contained polyhedral morphology, but non-co-culture group and PDLSCs demonstrated fibroblast-like cells (Fig. 3a). DNA methylations from non-co-culture and co-culture group were analyzed in C/EBPα, FABP4, LPL, and PPARγ2 genes using specific restriction enzymes digested at the cytocine phosphate guanine (CpG) sites (Fig. 4a). Digested and un-digested DNA fragments from all groups were verified the band intensities (Fig. 4b). All were compared DNA methylation status in mean of percentage ±SD. C/EBPα and LPL gene demonstrated statistically significant difference in methylation profiles after co-cultured, which increased from 47.12 ± 0.54 to 51.87 ± 0.58, and 66.9 ± 2.27 to 77.29 ± 0.11 (*P* < 0.01, 0.05), respectively. All genes from both groups except in FABP4 gene have shown statistically significant difference when compared with BMMSCs (P < 0.05) (Fig. 4c; Table 3). On the contrary, DNA methylation percentage of RUNX2 gene significantly reduced after co-cultured, which displayed 57.41 ± 2.16, 47.82 ± 2.9, and 47.04 ± 4 in non-co-culture, co-culture, and BMMSCs, respectively (Fig. 4d).

**Table 3:** DNA methylation in percentage of four adipogenic genes

Gene	Non-co-culture	Co-culture	BMMSCs
C/EBPα	47.12 ± 0.54	51.87 ± 0.58	27.17 ± 1.57
FABP4	61.71 ± 2.9	70.03 ± 12.41	66.53 ± 1.43
LPL	66.9 ± 2.27	77.29 ± 0.11	57.57 ± 3.62
PPARγ2	51.75 ± 4.23	56.77 ± 2.36	69.97 ± 1.83

Mean ± SD

### Osteogenic differentiation potential of DFAT cells is enhanced after PDLSCs co-culture

**Fig. 5:**
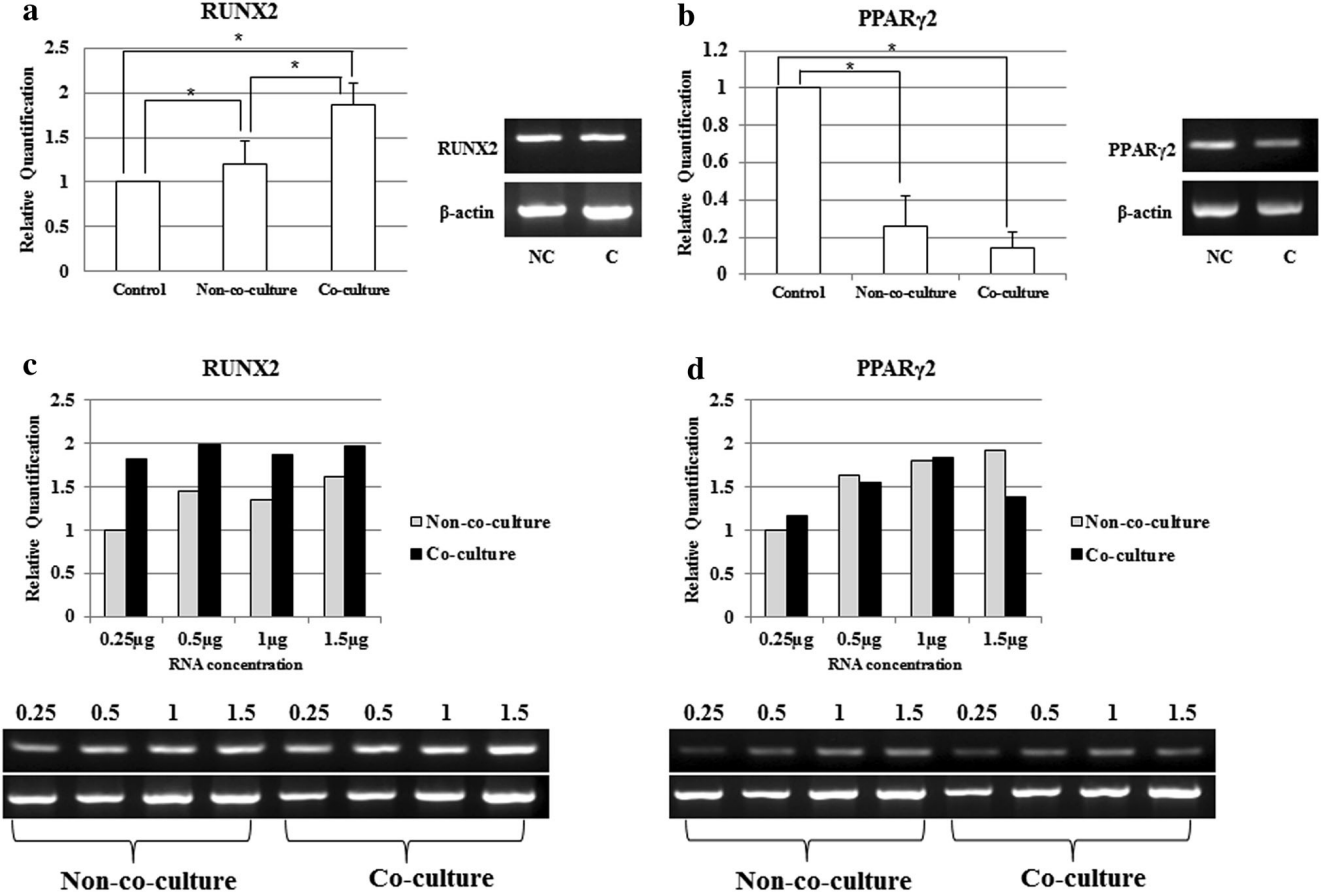
RUNX2 and PPARγ2 gene expression comparison by quantitative RT-PCR. After co-cultured for 2 weeks, osteogenic differentiation medium was replaced for induction the osteogenesis. **a**, **b** Real-time PCR was performed for analyzing the RUNX2 and PPARγ2 gene expression after osteogenic induction in co-culture DFAT cells comparing to non-co-culture and undifferentiated DFAT cells. There was statistically significant difference in RUNX2 gene expression level in co-culture group with in contrast of PPARγ2 gene expression level. **c**, **d** Semi-quantitative RT-PCR was analyzed in serial of RNA concentration. RUNX2 gene expression has up-regulated in all concentration of co-culture group. In contrast to PPARγ2, gene expression has down-regulated in co-culture group comparing to non-coculture group at 0.5 and 1.5 μg. *NC* means non-co-culture, *C* means co-culture. *Astrerisk* indicates that comparison was statistically significant difference at *P* < 0.05

After 2 weeks osteogenic induction, gene expression levels of RUNX2 and PPARγ2 gene were determined using real-time PCR normalizing by β-actin. RUNX2 gene expression significantly enhanced upon using co-culture system. It provided RUNX2 gene up-regulation higher than control and non-co-culture group (*P* < 0.05) (Fig. 5a). On the contrary, PPARγ2 gene expression level demonstrated attenuation profile in co-culture group. Unfortunately, data did not provide statistically significant difference when compared with non-co-culture group (Fig. 5b). For semi-quantitative RT-PCR, the relative band densities were evaluated with normalization by GAPDH in serial RNA concentration. Both genes also demonstrated the similar patterns to real-time PCR. RUNX2 gene in co-culture group was gradually increased by concentration (Fig. 5c). However, for PPARγ2 gene, co-culture group was expressed lower than non-co-culture group at 0.5 and 1.5 μg of RNA concentration (Fig. 5d).

## Discussion

Using cell-based therapy in periodontal regeneration, particularly from tooth-derived stem cells, is widely understood and has proven extremely potent for osteogenic differentiation. Even so, insufficient stem cell harvesting and high heterogeneity remain the limitations of PDLSCs. Consequently, other somatic stem cells have gained more attention for periodontal regeneration [3–6, 23–25]. Although DFAT cells derived from mature adipocytes are not stem cells, they provided homogeneity and a high expansion capability similar to that of other MSCs, such as BMMSCs and ASCs [12, 15]. Since they were an easily manipulated and abundant source, using DFAT cells in periodontal regeneration might be a novel source for cell-based therapy.

Our study first demonstrated the transcriptional and proliferative function, which was implied by DNA methylation profiles. All adipogenic genes, including C/EBPα, FABP4, LPL, and PPARγ2, increased DNA methylation in co-culture when compared with non-co-culture and control groups. Co-culture groups of all genes were indicated as hypermethylation, which was defined by methylation status more than 50 %. The hypermethylation status could down-regulate the transcriptional function, which resulted in silencing of the gene promoter and diminish in gene expression [26–28]. Our findings were consistent with a previous report that demonstrated an attenuated proliferative function in MSCs co-culture with three oral cells types but provided in low mitogenesis in the BrdU level than other gene expression activity [21]. On the other hand, our findings demonstrated that the methylation of RUNX2 gene established a converse effect to adipogenic genes in the co-culture group. The suggestion, therefore, was that the co-culture system might enhance the transcriptional function of RUNX2 gene.

In osteogenic differentiation, we examined RUNX2 and PPARγ2, which were gene expressions of osteogenic and adipogenic lineage. We compared these using real-time PCR and semi-quantitative RT-PCR after induction of an osteogenic differentiation medium. The DFAT cells co-culture displayed the greatest RUNX2 gene expression when compared with non-co-culture and control groups. Meanwhile, the PPARγ2 gene expression in the co-culture group demonstrated the lowest compared with the others. These indicated the linkage between the two contrary lineages in RUNX2 and PPARγ comparisons. Once RUNX2 is the preferable up-regulation, it drives the osteogenic differentiation and inhibits the PPARγ. The proposed manipulating this linkage using the transcriptional co-activator with a PDZ-binding motif or TAZ, which is the transcriptional co-activator used in RUNX2 for osteocalcin expression and PPARγ inhibition. TAZ plays the crucial role in binding with the 14-3-3 protein binding domain and the Pro-Pro-X-Tyr (PPXY) motif, which contains protein through the WW domain. Since both the RUNX2 and the PPARγ contain the PPXY domain, TAZ can interact with either RUNX2 or PPARγ to activate osteogenic differentiation while inhibiting PPARγ [22, 29–31]. Previous study demonstrated that transfected murine myoblast C2C12 cells by siRNA against TAZ isoform could inhibit the causal chain of osteoblastic differentiation via BMP-2 and osteocalcin gene expression by RUNX2 regulation. It was supposed that interaction of TAZ with RUNX2 effectively stimulated the osteocalcin gene promoter activity, a late marker of osteoblastic regeneration. On the other hand, TAZ binding to PPARγ could inhibit transcription from the aP2 gene promoter, which result in adipogenic differentiation down-regulation [22]. From these findings, we supposed that osteogenic and adipogenic lineages interacted oppositely due to a coordinating factor like TAZ.

RUNX2 and PPARγ are the pivotal transcriptional factors that can modulate MSCs into differentiating to the osteogenic or adipogenic lineage [32–35]. RUNX2 plays an essential role in osteoblastic differentiation and controls downstream target genes such as osteocalcin [36, 37]. It is possible to switch the MSCs for lineage differentiation depending upon the flavor factors and appropriate environment [22]. On the contrary, PPARγ is the key regulatory factor for adipogenic differentiation. A recent report has proposed a possible association between osteogenic and adipogenic differentiation that might be controlled via the signaling pathway of BMP4 and TNF-α. The PPARγ can suppress the effects of the BMP-type 2 receptor and the smad1/5/8 signaling thus resulting in adipogenic differentiation. At the same time, BMP4 and TNF-α could also down-regulate reversely to the PPARγ via the SAPK/JNK/NFκB/Stat signaling pathway, which provides the up-regulation of RUNX2 and osteogenesis [38].

The utility of the co-culture system has rarely been proposed regarding DFAT cells. A previous report demonstrated mature fat cells with endothelial cells co-culture. The histological feature of DFAT cells after co-culture demonstrated that the pre-adipocyte-like cells occurred in conjunction with DFAT cells and endothelial cells and generally expressed Flk-1, which was the endothelial cell marker. Moreover, DFAT cells also induced the endothelial cells by trans-differentiating into preadipocyte-like cells [20]. In terms of its osteogenic differentiation potential, the co-culture system could serve as an inductive process for PDLSCs when performed using endothelial cells. Several signaling molecules, including MEK/ERK, p38 MAPK, and COX-2/PGE2/VEGF in hypoxia condition enhanced osteogenic differentiation. While all factors of the osteogenic lineage demonstrated higher in co-culture versus non-co-culture groups, the effect of co-culture was not clearly identified, other than the hypoxia effects [19, 39].

Using adipose tissue as a cell-based procedure for periodontal regeneration has recently surfaced in the transplantation of ASCs in the oral rat model [23–25]. The role of DFAT cells in periodontal regeneration, however, was not investigated. Despite the presence of DFAT cells in the adipogenic lineage, they were enhanced by proper environment and they could be induced for osteogenic differentiation. This suggested that DFAT cells offered a potent function for differentiation. Therefore, DFAT cells could become a novel somatic cell source for periodontal regeneration that uses the co-culture system to enhance osteogenesis.

## Conclusion

Our finding first demonstrated the co-culture effect of DFAT cells with PDLSC in aspects of methylation profiles and in enhancing osteogenic gene expression. We also demonstrated the contrary effects between the osteogenic and adipogenic lineages through using the gene expression level. We concluded that DFAT cells might be an alternative cell-based therapy for periodontal regeneration.
